# Evolution of signalling systems with multiple senders and receivers

**DOI:** 10.1098/rstb.2008.0258

**Published:** 2008-12-12

**Authors:** Brian Skyrms

**Affiliations:** Department of Logic and Philosophy of Science, University of California Irvine3151 Social Science Plaza A, Irvine, CA 92717-5100, USA

**Keywords:** signalling, information, teamwork, population dynamics

## Abstract

Sender–receiver games are simple, tractable models of information transmission. They provide a basic setting for the study the evolution of meaning. It is possible to investigate not only the equilibrium structure of these games but also the dynamics of evolution and learning—with sometimes surprising results. Generalizations of the usual binary game to interactions with multiple senders, multiple receivers or both provide the elements of signalling networks. These can be seen as the loci of information processing, group decisions, and teamwork.

## 1. Introduction

To coordinate action, information must be transmitted, processed and used to make decisions. Transmission of information requires the existence of a signalling system in which the signals that are exchanged are coordinated with the appropriate content. Signalling systems in nature range from quorum signalling in bacteria (Schauder & Bassler 2001; Taga & Bassler 2003; Kaiser 2004), through the dance of the bees (Dyer & Seeley 1991), birdcalls (Hailman *et al*. 1985; Gyger *et al*. 1987; Evans *et al*. 1994; Charrier & Sturdy 2005) and alarm calls in many species (Cheney & Seyfarth 1990; Seyfarth & Cheney 1990; Green & Maegner 1998; Manser *et al*. 2002), to human language.

Information processing includes filtering—that is discarding irrelevant information and passing along what is important—and integration of multiple pieces of information. Filtering systems are ubiquitous. Quorum-sensing bacteria disregard low levels of signalling molecules, and only respond to concentrations appropriate to action. The black-capped chickadee *Poecile*, (*Poecile atricapilla*) disregards calls that lack the syntactic structure that identifies a chickadee origin. Every sensory processing system of a multicelled organism decides what information to discard and what to transmit. Integration includes computation, logical inference and voting. Although we usually think of these operations in terms of conscious human thought, they can also be performed unconsciously by simple signalling networks. Finally, information must be used to make decisions. These decisions may have fitness consequences for the whole group, down to the level of quorum sensing in bacteria and up to alarm calls and signals indicating location and quality of food sources.

From an evolutionary perspective, these three aspects of coordination are best addressed simultaneously. They may sometimes be separable in human affairs, but elsewhere in nature it is more typical that they have coevolved. It is possible to construct simplified models that capture essential aspects of these issues as evolutionary games.

These models may also be viewed as modules that, once evolved, may be put together to form more complex interactions. Evolutionary games may be studied from a both a static and dynamic point of view. Static analysis of equilibria reveals a lot about the structure of the interaction, and it can be carried out at a level of generality that does not commit one to a particular dynamics. But dynamic analysis sometimes reveals complexities that are not immediately apparent from the study of equilibria. Dynamic analyses may be mathematically challenging. Computer simulations are always available as a tool, but in these simple game-theoretic models, analytic methods are also applicable.

We start with dyadic sender–receiver games—one sender and one receiver—and then generalize the model to multiple senders and receivers. It can be shown that surprisingly sophisticated behaviour can emerge from the dynamics of evolution. A full analysis, however, is non-trivial in even the simplest dyadic signalling games, and much remains to be done.

## 2. Classic two-agent sender–receiver games: equilibrium considerations

In the basic model (Lewis 1969), there are two players: the sender and the receiver. Nature chooses a state with some probability (each state having non-zero probability of being chosen) and the sender observes the state. The sender then sends a signal to the receiver, who cannot observe the state directly but does observe the signal. The receiver then chooses an act, the outcome of which affects them both, with the pay-off depending on the state. We assume at the onset that the numbers of states, signals and acts are equal. Where this number is *N*, we refer to this as an *N*×*N*×*N* game.

There is pure common interest between sender and receiver—they get the same pay-off. There is exactly one ‘correct’ act for each state. In the correct act–state combination, they both get a pay-off of 1, otherwise pay-off is 0. We number the states and acts, so that in a play of the game, 〈state, signal, act〉=〈*s*_*i*_, *m*_*j*_, *a*_*k*_〉, the pay-off is 1 if *i*=*k*, 0 otherwise.

A sender's strategy consists of a function from states to signals and a receiver's from signals to acts. Expected pay-offs are determined by the probability with which nature chooses states and the population proportions of sender's and receiver's strategies. For the purposes of evolution, individual senders and receivers are assumed to have deterministic strategies.

Signals are not endowed with any intrinsic meaning. If they are to acquire meaning, the players must somehow find their way to an equilibrium where information is transmitted. When transmission is perfect, so that the act always matches the state and the pay-off is optimal, Lewis calls the equilibrium a *signalling system*. For instance, in a 3×3×3 game, the following combination of strategies is a Lewis signalling system equilibrium:

$$\begin{array}{cc} \text{sender} & \text{receiver}\\ \text{state}\;1\Rightarrow \text{signal}\;3 & \text{signal}\;3\Rightarrow \text{act}\;1\\ \text{state}\;2\Rightarrow \text{signal}\;2 & \text{signal}\;2\Rightarrow \text{act}\;2\\ \text{state}\;3\Rightarrow \text{signal}\;1 & \text{signal}\;1\Rightarrow \text{act}\;3 \end{array},$$

as is any combination of strategies that can be gotten from this one by permutation of signals. The ‘meaning’ of the signals is thus purely conventional, depending on the equilibrium into which the agents have settled.

There are also other equilibria in the signalling games. There are *pooling equilibria*, in which the sender ignores the state and the receiver ignores the signal. For example, suppose that state 3 is the most probable. Then, the following is a pooling equilibrium:

$$\begin{array}{cc} \text{sender} & \text{receiver}\\ \text{state}\;1\Rightarrow \text{signal}\;1 & \text{signal}\;3\Rightarrow \text{act}\;3\\ \text{state}\;2\Rightarrow \text{signal}\;1 & \text{signal}\;2\Rightarrow \text{act}\;3\\ \text{state}\;3\Rightarrow \text{signal}\;1 & \text{signal}\;1\Rightarrow \text{act}\;3 \end{array}.$$

Since the sender conveys no information, the receiver can do no better than choose the act that pays off in the most probable state. Since the receiver ignores the signal, the sender can do no better by changing his signalling strategy.

In *N*×*N*×*N* games with *N*>2, there are also *partial pooling equilibria*, for example,

$$\begin{array}{cc} \text{sender} & \text{receiver}\\ \text{state}\;1\Rightarrow \text{signal}\;3 & \text{signal}\;3\Rightarrow \text{act}\;1\\ \text{state}\;2\Rightarrow \text{signal}\;1 & \text{signal}\;2\Rightarrow \text{act}\;3\\ \text{state}\;3\Rightarrow \text{signal}\;1 & \text{signal}\;1\Rightarrow \text{act}\;3 \end{array}.$$

The sender's strategy does not discriminate between states 2 and 3 and leaves signal 2 unused. Upon receiving the ‘ambiguous’ signal, the receiver chooses optimally, given the limited information that was transmitted. For larger *N*, there are more kinds of partial pooling equilibria, depending on which states are ‘pooled’.

Among these equilibria, the signalling systems yield optimal pay-off, but this is no guarantee that one will arrive at them. They also, however, have the distinction of being *strict*; that is to say, any unilateral deviation results in a strictly worse pay-off. This has the immediate consequence that in an evolutionary setting, a signalling system is an *evolutionarily stable* state of the population. This is true both in a two-population evolutionary model, with a population of senders and receivers and in a one-population model in which an individual is sometimes in a sender role and sometimes in a position of being a receiver.

It is also easy to see that signalling systems are the *only* evolutionarily stable states (Wärneryd 1993). In the pooling example above, a mutant sender who always sent signal 2 would do just as well as the native population. Likewise, a mutant receiver whose strategy responded differently to the signal 3 (which is never sent) would not suffer for doing so. In the partial pooling example, a mutant sender who sent signal 2 in states 2 and 3 would elicit the same receiver response, and thus would have the same pay-off as the natives.

In each of these cases, the mutants do not do better than the natives. The pooling and partial pooling equilibria *are* equilibria. But the mutants do no worse, so they are not driven out. That is to say, pooling and partial pooling equilibria fail the test for evolutionary stability (Maynard Smith & Price 1973; Maynard Smith 1982). Equilibrium analysis might then lead one to suspect that evolutionary dynamics would always (or almost always) take us to signalling systems. It is not so (Huttegger 2007*a*,*b*, forthcoming; Pawlowitsch 2008).

## 3. Dynamics

The simplest dynamic model of differential reproduction for a large population is the *replicator dynamics* (Taylor & Jonker 1978; Hofbauer & Sigmund 1998). Replicator dynamics has an alternative interpretation as a model of cultural evolution by imitation of successful strategies (Björnerstedt & Weibull 1995; Schlag 1998). It has a third interpretation as a limiting case of reinforcement learning (Beggs 2005; Hopkins & Posch 2005).

We can consider a one-population model where strategies are conditional (if the sender does this, if the receiver does that), or a two-population model with one population of senders and another population of receivers. Both have biological applications. A two-population model is clearly appropriate for interspecies signalling. In case of same species alarm calls, individuals are sometimes in the role of sender and sometimes that of receiver.

For a single population, let the strategies be {*S*_*i*_}, let *x*_*i*_ be the population proportion of those who use strategy *S*_*i*_ and let the fitness of strategy *S*_*i*_ played against *S*_*j*_ be denoted *W*(*S*_*i*_|*S*_*j*_). Then, assuming random matching, the average fitness of strategy *S*_*i*_ is

$$W(S_{i})=\sum_{j}x_{j}W(S_{i}|S_{j}),$$

and the average fitness of the population is

$$W(S)=\sum_{i}W(S_{i})x_{i}.$$

The replicator dynamics is the system of differential equations

$$\frac{\text{d}x_{i}}{\text{d}t}=x_{i}[W(S_{i})-W(S)].$$

For the two-population case, let *x*_*i*_ be the population proportion of those who use strategy *S*_*i*_ in the population of senders and *y*_*i*_ be the population of those who use strategy *R*_*i*_ in the population of receivers. We again assume random matching of senders and receivers, so that

$$W(S_{i})=\sum_{j}y_{j}W(S_{i}|R_{j})\;\text{and}\;W(R_{j})=\sum_{i}x_{i}W(R_{j}|S_{i}).$$

The average fitnesses of the sender and receiver populations, respectively, are

$$W(S)=\sum_{i}W(S_{i})x_{i}\;\text{and}\;W(R)=\sum_{j}W(R_{j})y_{i}.$$

We consider the evolution of this two-population system using bipartite replicator dynamics (Taylor & Jonker 1978; Hofbauer & Sigmund 1998)

$$\frac{\text{d}x_{i}}{\text{d}t}=x_{i}[W(S_{i})-W(S)],$$

$$\frac{\text{d}y_{j}}{\text{d}t}=y_{j}[W(R_{j})-W(R)].$$

In both the one- and two-population models of Lewis' signalling games, the strong common interest between the sender and receiver assures *global convergence* of the replicator dynamics; all trajectories must lead to dynamic equilibria (Hofbauer & Sigmund 1998; Huttegger 2007*a*,*b*).

In the case of a 2×2×2 Lewis signalling game, with states equiprobable, the ‘hasty conclusion’ from evolutionary stability equilibrium analysis is, in fact, born out by the dynamics. Equilibria other than the signalling systems are all dynamically unstable. In both two- and one-population models, replicator dynamics carries almost all possible population proportions to a signalling system (Huttegger 2007*a*,*b*,*c*; Hofbauer & Huttegger 2008).

But if states are not equiprobable, this is no longer so. Suppose that state 2 is much more probable than state 1. Then, the receiver might just do the act that is the best in state 2 and ignore the signal. And since the signal is being ignored, the sender might as well ignore the state. Consider a population in which receivers always do act 2, some senders always send signal 1 and some always send signal 2. Any such population is an equilibrium. We have described a set of polymorphic pooling equilibria. These equilibria are dynamically stable, even though they are not evolutionarily stable in the sense of Maynard-Smith & Price (1973). They are not strongly stable attractors in the dynamics. Rather, they are ‘neutrally stable’, in that points near them stay near them under the action of the dynamics. But they do not attract all points near them. For instance, other pooling equilibria near them are not moved at all by the dynamics. The question is whether this set of pooling equilibrium, considered as a whole, has a basin of attraction. It has been shown analytically that it does (Hofbauer & Huttegger 2008). Simulations show that the size of the basin of attraction need not be negligible. The size depends, as would be expected, on the difference in the probabilities of the two states. If we were to depart from the assumption that the states have equal pay-offs, it would also depend on the magnitudes of the pay-offs.

Even if we keep the states equiprobable and the magnitudes of the pay-offs equal, almost sure convergence to a signalling system is lost as we move from 2×2×2 to 3×3×3. In this game, total pooling equilibria are dynamically unstable, but there are sets of neutrally stable partial pooling equilibria as the ones discussed in the last section. It can be shown analytically that the set of partial pooling equilibria has a positive basin of attraction, and simulation shows that this basin is not negligible (Huttegger *et al*. in press).

Even with the strong common interest assumptions built into Lewis' signalling games, the emergence of signalling is not quite the sure thing that it may initially have seemed on the basis of equilibrium considerations. Perfect signalling systems can evolve, but it is not guaranteed that they will do so. Dynamic analysis has revealed unexpected subtleties.

There are more subtleties to explore, because the sets of suboptimal equilibria are not *structurally stable* (Guckenheimer & Holmes 1983; Skyrms 1999) Small perturbations of the dynamics can make a big difference. The natural perturbation to pure differential reproduction that needs to be considered is the addition of a little mutation. We can move from the replicator dynamics to the replicator–mutator dynamics (Hadeler 1981; Hofbauer 1985). For a two-population model with uniform mutation, this is

$$\frac{\text{d}x_{i}}{\text{d}t}=x_{i}[(1-e)W(S_{i})-W(S)]+(e/n)W(S),$$

$$\frac{\text{d}y_{j}}{\text{d}t}=y_{j}[(1-e)W(R_{j}-W(R)]+(e/n)W(R),$$

where *e* is the mutation rate and *n* is the number of strategies. We include all possible strategies. Evolutionary dynamics is now governed by a sum of selection and mutation pressures. Mutation pressure pushes towards all strategies being equiprobable, where mutation into a strategy would equal mutation out. Mutation pressure can be counterbalanced or overcome by selection pressure. But if selection pressure is weak or non-existent, mutation can cause dramatic changes in the equilibrium structure of the interaction.

We can illustrate by returning to the 2×2×2 signalling game, two-population states with unequal probability. Suppose state 2 is more probable than state 1. Then, as we have seen, there is a set of pooling equilibria for the replicator dynamics. In the receiver population, the strategy of always doing act 2 (no matter what the state is) goes to fixation. In the sender population, there is a polymorphism between two types of sender. One sends signal 1, no matter what the state is; the other sends signal 2, no matter what the state is. Since there is no selection pressure between the senders' types, every such sender polymorphism is an equilibrium. Addition of *any* amount of uniform mutation leads the set of pooling equilibria to collapse to a single point at which ‘Always send signal 1’ and ‘Always send signal 2’ are represented with equal probability (Hofbauer & Huttegger 2008). But all other strategies are also present in small amounts at this population state, due to the action of mutation.

The big question concerns the stability properties of this *perturbed pooling equilibrium*. Is it dynamically stable or unstable? There is no unequivocal answer. It depends on the disparity in the probability between the two states (Hofbauer & Huttegger 2008). A little mutation can help the evolution of signalling systems, but does not always guarantee that they evolve.

## 4. Costs

Let us return to the case of 2×2×2, with states equiprobable, but assume that *one of the signals costs something to send, while the other is cost free*. (We could interpret the cost-free signal as just keeping quiet.) Now there are pooling equilibria in which the sender always sends the cost-free signal and there are various proportions of receiver types.

Denoting the sender's strategies as

$$\begin{array}{cc} \text{sender}\;1:\text{state}\;1\Rightarrow \text{signal}\;1, & \text{state}\;2\Rightarrow \text{signal}\;2\\ \text{sender}\;2:\text{state}\;1\Rightarrow \text{signal}\;2, & \text{state}\;2\Rightarrow \text{signal}\;1\\ \text{sender}\;3:\text{state}\;1\Rightarrow \text{signal}\;1, & \text{state}\;2\Rightarrow \text{signal}\;1\\ \text{sender}\;4:\text{state}\;1\Rightarrow \text{signal}\;2, & \text{state}\;2\Rightarrow \text{signal}\;2 \end{array}$$

and the receiver's strategies as

$$\begin{array}{cc} \text{receiver}\;1:\text{signal}\;1\Rightarrow \text{act}\;1, & \text{signal}\;2\Rightarrow \text{act}\;2\\ \text{receiver}\;2:\text{signal}\;1\Rightarrow \text{act}\;2, & \text{signal}\;2\Rightarrow \text{act}\;1\\ \text{receiver}\;3:\text{signal}\;1\Rightarrow \text{act}\;1, & \text{signal}\;2\Rightarrow \text{act}\;1\\ \text{receiver}\;4:\text{signal}\;1\Rightarrow \text{act}\;2, & \text{signal}\;2\Rightarrow \text{act}\;2 \end{array}.$$

If signal 1 is costly, cost=2*c*, states equiprobable and a background fitness is 1, we have the pay-off matrix (sender's pay-off, receiver's pay-off), as shown in table 1.

Sender's strategies 1 and 2 pay the cost half the time, strategy 3 all the time and strategy 4 never. Pure Nash equilibria of the game for small *c* are italic-faced. (If *c*>0.5, it is never worth the cost to send a signal, and the signalling system equilibria disappear.) There is also a large range of mixed strategies (corresponding to the receiver polymorphisms) that are equilibria. States when receiver types are approximately equally represented and senders always send the costless signal are such pooling equilibria.

It might also *cost the receiver something to listen*. Let us combine this with a costly message and unequal state probabilities. For example, let the probability of state 1 be 1/3, the cost of signal 1 0.3 and the cost of the receiver paying attention to the signals 0.1. The background fitness is 1. Then, the foregoing pay-off matrix changes to that displayed in table 2.

The *pooling equilibrium*, 〈sender 4, receiver 4〉, where the sender always sends signal 2 and the receiver always does act 2, is now a *strict* Nash equilibrium of the game. Either the sender or receiver who deviates does strictly worse. Thus, in both one- and two-population evolutionary models, it is *evolutionarily stable* and a strong (attracting) equilibrium in the replicator dynamics.

*If costs are state specific*, a rosier picture is possible (Zahavi 1975). We alter the previous example so that signal 1 is free in state 1 but costs 0.3 in state 2 and signal 2 is free in state 2 but costs 0.3 in state 1. Sender 1 now pays no penalty; sender 2 always pays 0.3; sender 3 pays 0.3 two-thirds of the time (=0.2) and sender 4 pays 0.3 one-third of the time (=0.1). This is shown in table 3.

The pooling state, 〈sender 4, receiver 4〉, is no longer an equilibrium at all. Given that the receiver is ignoring the message, the sender is better off switching to the costless strategy, sender 1. If so, the receiver is better off switching to receiver 1, yielding the optimal signalling system 〈sender 1, receiver 1〉. Optimality, however, may not evolve. The *suboptimal signalling system* 〈sender 2, receiver 2〉, in which the sender uses the ‘wrong’ signals and always pays a signalling cost, is also a strict equilibrium. Both signalling systems are strong (attracting) equilibria in both one- and two-population replicator dynamic models.

## 5. Signalling networks

There is no reason to limit ourselves to signalling between just two actors: one sender and one receiver. In fact, most signalling systems in nature involve multiple senders, multiple receivers or both. If a receiver gets signals carrying different pieces of information from different senders, the signalling system is called upon to solve some problem of information processing. Consider a toy model with two senders and one receiver

•→•←•

*Signalling complementary information*. There are four states of nature, each of which occurs with non-zero probability. Two individuals are situated so as to make different incomplete observations of the state. The first sees whether it is in {S1, S2} or in {S3, S4} and the second sees whether it is in {S1, S3} or in {S2, S4}. Together, they have enough information to pin down the state of nature, but separately they do not. Each sends one of two signals to a receiver who must choose one of four acts. Let us say the first sender chooses ‘red’ or ‘green’ and the second chooses ‘blue’ or ‘yellow’. The pay-offs favour cooperation. Exactly one act is ‘right’ for each of the states, in that each of the individuals is reinforced just in case the right act for the state is chosen.

In this extended Lewis signalling game, the observational situation of sender 1 is characterized by a partition of the states, *O*_1_={{S1, S2}, {S3, S4}}. Her signalling strategy is a function from the elements of this partition into her set of signals, {R, G}. Likewise sender 2 in observational situation O_2_={{S1, S3}, {S2, S4}} has a signalling strategy that maps the elements of her partition into her signal set, {B, Y}. The receiver's strategy maps pairs of signals {{R, B}, {R, Y}, {G, B}, {G, Y}} into her set of acts {A1, A2, A3, A4}.

All agents get pay-off 1 just in case the receiver correctly identifies the state and does the appropriate act. Pay-offs are shown in table 4.

A *signalling system* equilibrium is a combination of sender and receiver strategies such that pay-off is equal to 1 in each state. As before, a signalling system is a *strict equilibrium* of the game, and the signalling systems are the *only* strict equilibria. There are lots of pooling and partial pooling equilibria.

In an evolutionary setting, this three-player game gives rise to three-, two- and one-population models. In a one-population model, an individual's strategy would be of the form: *if sender in observational situation* *O*_1_ *has this sender's strategy; if sender in observational situation* *O*_2_ *has that sender's strategy; and if receiver has this strategy.* The most natural two-population model has a population of senders with different observational roles and a population of receivers. In all three evolutionary settings, signalling systems are the unique evolutionarily stable states. It is no longer certain that a signalling system must evolve, but it is certain that a signalling system *can* evolve. In each of these settings, a signalling system is a strongly stable (attracting) equilibrium in the replicator dynamics.

Each sender's signal conveys perfect information about her observation—about the partition of the states of the world which she can see. The combination of signals has perfect information about the states of the world. Exactly one state corresponds to each combination of signals. And the receiver puts the signals together. The receiver's acts contain perfect information about the state of the world. *The signalling system simultaneously solves problems of transmission and integration of information*.

The basic model admits of interesting variations. Of course, there may be more senders. And depending on the act set available to the receiver, he may draw the appropriate logical ‘conclusion’ from the ‘premises’ supplied by the various senders (Skyrms 2000, 2004, 2008). The senders' partitions may not be fixed by nature, but may themselves evolve in the presence of information bottlenecks (Barrett 2006, 2007*a*,*b*).

*Error*: There is another class of multiple sender models, where the question is not one of complementary information but one of error. In the previous example, senders observed different partitions but there was no error in identifying the true element of the partition. Here, we suppose that the senders all observe the same states but with some error in correctly identifying them. (An alternative, essentially equivalent, interpretation of the model would locate the errors in the transmission of the signals.)

For the simplest model, suppose that there are only two states and two acts. States are equiprobable. Three senders observe the states with error probability of 10 per cent, with the errors being independent between senders and between trials. Each sender sends a message to the receiver, who must then choose one of the two acts. As before, we assume that act 1 pays off 1 for everyone involved in state 1 and act 2 pays off 1 for everyone involved in state 2. Otherwise, no one gets anything.

Nature here first flips a coin to pick a state, and then picks *apparent states* to present to the three senders according to the error probabilities. A sender's strategy is a function from apparent state into the set of signals, {S1, S2}. We have a choice about how to set up the receiver's strategies. If we were to assume that the receiver could distinguish between senders, we could take the receiver's strategy to be a function from ordered triples of signals to acts. But here we assume that the receiver cannot distinguish between 〈S1, S2, S1〉, 〈S1, S1, S2〉 and 〈S1, S1, S2〉. The receiver here has an observational partition and can only count signals. This might be thought of as discrete approximation to a situation where the receiver perceives an intensity arising from many chemical signals, or the sound intensity arising from many calls. A receiver's strategy is then a function from the frequencies of signal received to act.

Optimal signalling in this model consists in what we might call a *Condorcet equilibrium* (see List *et al*. 2009; Sumpter & Pratt 2009). There is one signal that the senders all use for apparent state 1 and another that they all use for apparent state 2. The receiver goes with a majority vote. For instance, if the senders all send signal 2 in state 1, the receiver will do act 2 if two or more senders send signal 2 and act 1 otherwise. In our example, individuals at a Condorcet equilibrium reduce their error rate from 10 per cent to under 3 per cent. This can be viewed as an example of information filtering, as explained in §1.

Rather than thinking of evolution taking place solely in the context of this game, we might assume that sender's strategies already evolved in the context of single sender–receiver interactions. Then, receivers usually get one signal, or multiple agreeing signals according to the evolved signalling system, but occasionally get disagreeing signals. Slow adaptation for mixed signals in such an environment is a simple problem of optimization.

Against these fixed sender strategies, receivers who go with the majority of senders will have the greatest fitness. Then replicator dynamics will converge to the optimal receiver strategy (Hofbauer & Sigmund 1998).

But suppose we forego this easy route and ask whether Condorcet signalling equilibria can evolve in the context of the original four-person game. Both the sender's signals and the receiver's voting rule must coevolve. It is still possible for efficient signalling to evolve. The Condorcet equilibria are strict. Consequently, they are stable attractors in the evolutionary versions of this game using replicator dynamics. In fact, simulations show the Condorcet equilibria almost always evolving in the foregoing model (see the electronic supplementary material).

Variations in the parameters of the model may well lead to the evolution of voting rules different from majority rule. This is an area open for exploration. Recent rational-choice literature on strategic voting (Austen-Smith & Banks 1996; Feddersen & Pesendorfer 1998) is a source of a rich set of models that can be transposed to an evolutionary setting.

*Teamwork*: It is sometimes the case that a well-placed sender knows what needs to be done, and can send messages to receivers who can act, but that no one receiver can do everything that needs to be done. The sender may be the foreman, the commander or the brain of an organism—the team leader. Success for all requires teamwork.

There may be one sender and multiple receivers

•←•→•

For a simple teamwork problem, we suppose that there are two receivers and one sender. The sender observes one of four equiprobable states of the world and sends one of two signals to each receiver. The receivers must each choose between two acts, and the acts must be coordinated in a way determined by the state for all to get a pay-off. We take pay-offs to be as shown in table 5.

We assume that the sender can distinguish the members of the team; so the sender's strategy maps states into ordered pairs of signals and a receiver's strategy maps her signal into her space of acts. Here, the problem to be solved is a combination of one of communication and one of coordination. It is solved in a signalling system equilibrium, in which everyone always gets pay-off of 1. A signalling system equilibrium is again a strict equilibrium, and the unique strict equilibrium in the game. It is a strongly stable attractor in the replicator dynamics.

The example can be varied in many ways, some more interesting than others. The two receivers can be thought of as playing a rather trivial two-person game, but the game is different in every state of the world. In a signalling system, the sender can be thought of either as conveying information about the game or the optimal act to be done. In these trivial games, these are equivalent. The example could be varied by changing the four embedded two-person games and their effect on the pay-offs to the sender.

*Chains*: Information can flow further than that shown in the models given so far. Signallers can form chains, so that information is passed along until it reaches an endpoint at which it can be used. Consider a little signalling chain

•→•→•

There is a sender, an intermediary and a receiver. Nature chooses one of two states with equal probability. The sender observes the state, chooses one of two signals and sends it to the intermediary, the intermediary observes the sender's signal, chooses one of her own two signals and sends it to the receiver. The receiver observes the intermediary's signal and chooses one of two acts. If the act matches the state, sender, intermediary and receiver all get a pay-off of 1, otherwise a pay-off of 0.

Suppose that the set of potential signals available to the sender is {R, B}, and that available to the receiver is {G, Y}. A sender's strategy is a function from {S1, S2} into {R, B}, an intermediary's from {R, B} into {G, Y} and a receiver's from {G, Y} into {A1, A2}. A signalling system here is a triple of strategies such that the composition of sender's, intermediary's and receiver's strategies maps state 1 to act 1 and state 2 to act 2. Signalling systems are the unique strict equilibria in this game and the unique evolutionarily stable states in the corresponding one-, two- and three-population signalling games. They are attractors in the replicator dynamics. In principle, signalling chains can evolve out of nothing.

However, simulations show that in this case evolution is very slow when compared with the other signalling games discussed so far. This may simply be a consequence of the multiplicity of coordination problems that need to be solved simultaneously. The speed with which the chain signalling system can evolve is much improved if the sender and receiver have pre-existing signalling systems. They could be the same signalling system, which would be plausible if the sender and receiver were the members of the same population, but the signalling systems need not be the same. The sender and receiver can have different ‘languages’, so that the intermediary has to act as a ‘translator’ or signal transducer. Suppose that the sender sends red or blue and the ultimate receiver reacts to green or yellow as follows:

$$\begin{array}{cc} \text{sender} & \text{receiver}\\ \text{state}\;1\Rightarrow \text{R} & \text{G}\Rightarrow \text{act}\;2\\ \text{state}\;2\Rightarrow \text{B} & \text{Y}\Rightarrow \text{act}\;1 \end{array}.$$

A successful translator must learn to receive one signal and send another, so that the chain leads to a successful outcome.

$$\begin{array}{ccc} \text{sender} & \text{translator} & \text{receiver}\\ \text{state}\;\text{1}\Rightarrow \text{R} & \text{see}\;\text{R}\Rightarrow \text{send}\;\text{Y} & \text{Y}\Rightarrow \text{act}\;\text{1}\\ \text{state}\;\text{2}\Rightarrow \text{B} & \text{see}\;\text{B}\Rightarrow \text{send}\;\text{G} & \text{G}\Rightarrow \text{act}\;\text{2} \end{array}.$$

The translator's learning problem is now really quite simple. The requisite strategy strictly dominates all alternatives. It pays off all the time, while the strategies *always send Y* and *always send G* pay off half the time, and the remaining possibility always leads to failure. The dominated strategies are eliminated (Hofbauer & Sigmund 1998), and the correct strategy evolves.

*Dialogue*: The chain model showed one way in which simple interactions could be strung together to form more complex signalling systems. Here is another. Suppose that a sender's observational partition is not fixed. The sender can choose which observation to make. That is to say, she can choose which partition of states to observe. Suppose also that the receiver's decision problem is not fixed. Nature chooses a decision problem to present to the receiver. Different sorts of information are relevant to different decision problems. Knowing the actual element of partition A (the element that contains the actual state) may be relevant to decision problem 1, while knowing the actual element of partition B may be relevant to decision problem 2. This opens up the possibility of signalling dialogue, where information flows in two directions

•↔•

In the simplest sort of example, nature flips a coin and presents player 2 with one or another decision problem. Player 2 sends one of two signals to player 1. Player 1 selects one of two partitions of the state of nature to observe. Nature flips a coin and presents player 1 with the true state. Player 1 sends one of two signals to player 2. Player 2 chooses one of two acts.

Suppose that there are four states, {S1, S2, S3, S4}, with alternative partitions: P1={{S1, S2}, {S3, S4}}, P2={{S1, S3}, {S2, S4}}. The two decision problems require choices in different act sets: D1= {A1, A2}, D2={A3, A4}. Pay-offs for the two decision problems are shown in table 6.

Player 2 has a signal set {R, G} and player 1 has a signal set {B, Y}. A strategy for player 2 now consists of three functions: a sender strategy from {P1, P2} into {R, G}; a receiver strategy form {B,Y} into {A1, A2}; and a receiver strategy from {B, Y} into {A3, A4}. In a signalling system equilibrium, each player gets always a pay-off of 1. The possibility of dialogue introduces a plasticity of signalling that is absent in fixed sender–receiver games. Signalling systems are strict and evolutionarily stable as before.

Signalling systems can evolve in the dialogue interaction in isolation, but simulations show this process to be very slow. As in the case of chains, evolution of a signalling system is much easier if we assume that some of its parts have evolved in less complicated interactions. Player 1 may already have signalling systems in place for the two different observational partitions as a consequence of evolution in simple sender–receiver interactions. If so, the evolution of dialogue only requires that the second player signals the problem and the first chooses what to observe. This is no more difficult than the evolution of a signalling system in the original Lewis signalling game.

## 6. Conclusion

We have investigated the evolution of signalling in some modest extensions of Lewis signalling games with multiple senders and receivers. This discussion has focused on one particular setting—a large (infinite) population or several large populations with random interactions between individuals. Different settings would call for different relevant dynamics. A small population with random encounters calls for a stochastic model of evolution, with either a growing population or one whose size is fixed at some carrying capacity (Shreiber 2001; Benaim *et al*. 2004; Taylor *et al*. 2004). Pawlowitsch (2007) has shown that in one kind of finite population model, efficient proto-languages are the only strategies that are *protected by selection*. Individuals might interact with neighbours in some spatial structure (Grim *et al*. 2002; Zollman 2005). Isolated individuals might invent signalling systems by trial-and-error learning in repeated interactions (Skyrms 2004, 2008; Barrett 2006, 2007*a*,*b*), which might then spread by a process of cultural evolution (Komarova & Niyogi 2004). In fact, urn models of reinforcement learning are very close to those in a small, growing population (Shreiber 2001; Benaim *et al*. 2004). It has been recently proved that reinforcement dynamics in the simplest Lewis signalling game—2×2×2 states equiprobable—converges with probability 1 to a signalling system (Argiento *et al*. in press). Such an analytic treatment of reinforcement learning does not yet exist for more complicated signalling interactions, but simulations tend to give results parallel to the evolutionary analysis given here. This is not entirely surprising, given the close connections between reinforcement learning and the replicator dynamics (Beggs 2005; Hopkins & Posch 2005).

Simple models such as those discussed here can be assembled into more complex and biologically interesting systems. The network topologies themselves may evolve (Bala & Goyal 2000; Skyrms & Pemantle 2000). There are all sorts of interesting variations. For instance, signalling networks may allow eavesdroppers, a case well studied in (McGregor 2005). But the main business of signalling networks is to facilitate successful collective action. The simple models studied here focus on the crucial aspects of coordinated action. Information is acquired by the units of the group. It is transmitted to other units and processed in various ways. Extraneous information is discarded. Various kinds of computation and inference are performed. The resulting information is used to guide group decisions that lead to coordinated action. All this can happen either with or without conscious thought. These processes are instantiated in human organizations, in the coordination of the organs and cells of a multicellular organism and even within the cells themselves. Information flows through signalling networks at all levels of biological organization.

## Figures and Tables

**Table 1:** Pay-offs if sending signal is costly.

	receiver 1	receiver 2	receiver 3	receiver 4
sender 1	*2*-c, *2*	1-*c*, 1	1.5-c, 1.5	1.5-c, 1.5
sender 2	1-c, 1	*2*-c, *2*	1.5-c, 1.5	1.5-c, 1.5
sender 3	1.5-2c, 1.5	1.5-2c, 1.5	1.5-2c, 1.5	1.5-2c, 1.5
sender 4	1.5, 1.5	1.5, 1.5	*1.5, 1.5*	*1.5, 1.5*

**Table 2:** Pay-offs if receiving signal is costly.

	receiver 1	receiver 2	receiver 3	receiver 4
sender 1	*2-0.1, 2-0.1*	1-0.1, 1-0.1	1.33-0.1, 1.33	1.67-0.1, 1.67
sender 2	1-0.2, 1-0.1	*2-0.2, 2-0.1*	1.33-0.2, 1.33	1.67-0.2, 1.67
sender 3	1.5-0.3, 1.5-0.1	1.5-0.3, 1.5-0.1	1.33-0.3, 1.33	1.67-0.3, 1.67
sender 4	1.5, 1.5-0.1	1.5, 1.5-0.1	1.33, 1.33	*1.67, 1.67*

**Table 3:** Pay-offs if costs are state specific.

	receiver 1	receiver 2	receiver 3	receiver 4
sender 1	*2, 2-0.1*	1, 1-0.1	1.33, 1.33	1.67, 1.67
sender 2	1-0.3, 1-0.1	*2-0.3, 2-0.1*	1.33-0.3, 1.33	1.67-0.3, 1.67
sender 3	1.5-0.2, 1.5-0.1	1.5-0.2, 1.5-0.1	1.33-0.2, 1.33	1.67-0.2, 1.67
sender 4	1.5-0.1, 1.5-0.1	1.5-0.1, 1.5-0.1	1.33-0.1, 1.33	1.67-0.1, 1.67

**Table 4:** Pay-offs with two senders and one receiver.

	act 1	act 2	act 3	act 4
state 1	1,1,1	0,0,0	0,0,0	0,0,0
state 2	0,0,0	1,1,1	0,0,0	0,0,0
state 3	0,0,0	0,0,0	1,1,1	0,0,0
state 4	0,0,0	0,0,0	0,0,0	1,1,1

**Table 5:** Pay-offs in a simple teamwork situation.

	〈A1, A1〉	〈A1, A2〉	〈A2, A1〉	〈A2, A2〉
state 1	1,1,1	0,0,0	0,0,0	0,0,0
state 2	0,0,0	1,1,1	0,0,0	0,0,0
state 3	0,0,0	0,0,0	1,1,1	0,0,0
state 4	0,0,0	0,0,0	0,0,0	1,1,1

**Table 6:** Pay-offs in a dialogue situation.

	decision 1	decision 1	decision 2	decision 2
	act 1	act 2	act 3	act 4
state 1	1	0	1	0
state 2	1	0	0	1
state 3	0	1	1	0
state 4	0	1	0	1

## Supplementary Material

Supporting matter for evolution of signaling systems with multiple senders and receivers
