# Supplementary material for: Evolution of signalling systems with multiple senders and receivers
Source: Philos Trans R Soc Lond B Biol Sci. 2008 Dec 12;364(1518):771–9. doi: 10.1098/rstb.2008.0258 (PMC2689717; doi:10.1098/rstb.2008.0258)
Supplement: Supporting matter for evolution of signaling systems with multiple senders and receivers [file rstb20080258s01.doc]

# Evolution of Signaling Systems with Multiple Senders and Receivers

**Brian Skyrms**^1^

*^1^Department of Logic and Philosophy of Science, University of California Irvine, 3151 Social Science Plaza A, Irvine, CA. 92717-5100 USA, bskyrms@uci.edu*

Sender-Receiver games are simple, tractable models of information transmission. They provide a basic setting for the study the evolution of meaning. It is possible to investigate not only the equilibrium structure of these games, but also the dynamics of evolution and learning – with sometimes surprising results. Generalizations of the usual binary game to interactions with multiple senders, multiple receivers, or both, provide the elements of signaling networks. These can be seen as the loci of information processing, of group decisions, and of teamwork.

**Keywords:** signaling, information, teamwork, population dynamics

**1. Introduction**

To coordinate action, information must be transmitted, processed, and utilized to make decisions. Transmission of information requires the existence of a signaling system in which the signals that are exchanged are coordinated with the appropriate content. Signaling systems in nature range from quorum signaling in bacteria (Schauder & Bassler 2001; Kaiser 2004), through the dance of the bees (Dyer & Seeley 1991), birdcalls (Hailman *et al.* 1985; Gyger *et al.* 1987; Evans *et al.* 1994; Charrier & Sturdy 2005), and alarm calls in many species (Seyfarth & Cheney 1990; Green & Maegner 1998; Manser *et al.*2002), up to human language.

Information processing includes filtering – that is discarding irrelevant information and passing along what is important – and integration of multiple pieces of information. Filtering systems are ubiquitous. Quorum-sensing bacteria disregard low levels of signaling molecules, and only respond to concentrations appropriate to action. The black-capped chickadee disregards calls which lack the syntactic structure that identifies a chickadee origin. Every sensory processing system of a multi-celled organism decides what information to discard and what to transmit. Integration includes computation, logical inference, and voting. Although we usually think of these operations in terms of conscious human thought, they can also be performed unconsciously by simple signaling networks. Finally, information must be used to make decisions. These decisions may have fitness consequences for the whole group, down to the level of quorum sensing in bacteria and up to alarm calls and signals indicating location and quality of food sources.

From an evolutionary perspective, these three aspects of coordination are best addressed simultaneously. They may sometimes be separable in human affairs, but elsewhere in nature it is more typical that they have coevolved. It is possible to construct simplified models which capture essential aspects of these issues as evolutionary games.

These models may also be viewed a modules that, once evolved, may be put together to form more complex interactions. Evolutionary games may be studied from a both a static and a dynamic point of view. Static analysis of equilibria reveals a lot about the structure of the interaction, and it can be carried out at a level of generality that does not commit one to a particular dynamics. But dynamic analysis sometimes reveals complexities that are not immediately apparent from the study of equilibria. Dynamic analyses may be mathematically challenging. Computer simulations are always available as a tool, but in these simple game-theoretic models analytic methods are also applicable.

We start with dyadic sender-receiver games – one sender and one receiver – and then generalize the model to multiple senders and multiple receivers. It can be shown that surprisingly sophisticated behavior can emerge from dynamics of evolution. A full analysis, however, is non-trivial in even the simplest dyadic signaling games, and much remains to be done.

**2. Classic Two-Agent Sender-Receiver Games: Equilibrium Considerations**

In the basic model (Lewis 1969), there are two players, the sender and the receiver. Nature chooses a state with some probability (each state having non-zero probability of being chosen) and the sender observes the state. The sender then sends a signal to the receiver, who cannot observe the state directly but does observe the signal. The receiver then chooses an act, the outcome of which affects them both, with the payoff depending on the state. We assume at the onset that the numbers of states, signals and acts are equal. Where this number is N, we refer to this as an NbyNbyN game.

There is pure common interest between sender and receiver– they get the same payoff. There is exactly one “correct” act for each state. In the correct act-state combination they both get a payoff of one; otherwise payoff is zero. We number the states and acts so that in a play of the game, <state, signal, act> = <s_i_, m_j_, a_k_> the payoff is

1 if i=k, 0 otherwise.

A sender’s strategy consists of a function from states to signals; a receiver’s strategy consists of a function from signals to acts. Expected payoffs are determined by the probability with which nature chooses states, and the population proportions of sender’s and receiver’s strategies. For the purposes of evolution, individual senders and receivers are assumed to have deterministic strategies.

Signals are not endowed with any intrinsic meaning. If they are to acquire meaning, the players must somehow find their way to an equilibrium where information is transmitted. When transmission is perfect, so that the act always matches the state and the payoff is optimal, Lewis calls the equilibrium a *signaling system*. For instance, in a 3by3by3 game the following combination of strategies is a Lewis signaling system equilibrium:

SENDER RECEIVER

State 1 => Signal 3 Signal 3 => Act 1

State 2 => Signal 2 Signal 2 => Act 2

State 3 => Signal 1 Signal 1 => Act 3

as is any combination of strategies that can be gotten from this one by permutation of signals. The “meaning” of the signals is thus purely conventional, depending on the equilibrium into which the agents have settled.

There are also other equilibria in signaling games. There are *pooling equilibria*, in which the sender ignores the state, and the receiver ignores the signal. For example, suppose that state 3 is the most probable. Then the following is a pooling equilibrium:

SENDER RECEIVER

State 1 => Signal 1 Signal 3 => Act 3

State 2 => Signal 1 Signal 2 => Act 3

State 3 => Signal 1 Signal 1 => Act 3

Since the sender conveys no information, the receiver can do no better than choose the act that pays off in the most probable state. Since the receiver ignores the signal, the sender can do no better by changing his signaling strategy.

In NbyNbyN games with N>2, there are also *partial pooling equilibria*, for example:

SENDER RECEIVER

State 1 => Signal 3 Signal 3 => Act 1

State 2 => Signal 1 Signal 2 => Act 3

State 3 => Signal 1 Signal 1 => Act 3

The sender’s strategy does not discriminate between states 2 and 3, and leaves signal 2 unused. Upon receiving the “ambiguous” signal, the receiver chooses optimally given the limited information that was transmitted. For larger N, there are more kinds of partial pooling equilibria, depending on which states are “pooled.”

Among these equilibria, signaling systems yield optimal payoff, but this is no guarantee that one will arrive at them. They also, however, have the distinction of being *strict*, that is to say that any unilateral deviation results a strictly worse payoff. This has the immediate consequence that in an evolutionary setting a signaling system is an *evolutionarily stable* state of the population. This is true both in a 2-population evolutionary model, with a population of senders and a population of receivers and in a one population model in which an individual is sometimes in a sender role and sometimes in a position of being a receiver.

It is also easy to see that signaling systems are the *only* evolutionarily stable states. (Wärneryd 1993) In the pooling example above, a mutant sender who always sent signal 2 would do just as well as the native population. Likewise, a mutant receiver whose strategy responded differently to the signal 3 (which is never sent) would not suffer for doing so. In the partial pooling example, a mutant sender who sent signal 2 in states 2 and 3 would elicit the same receiver response, and thus would have the same payoff as the natives.

In each of these cases, the mutants do not do better than the natives. The pooling and partial pooling equilibria *are* equilibria. But the mutants do no worse, so they are not driven out. That is to say that pooling and partial pooling equilibria fail the test for evolutionary stability (Maynard Smith and Price 1973). Equilibrium analysis might then lead one to suspect that evolutionary dynamics would always (or almost always) take us to signaling systems. It is not so. (Pawlowitsch 2008; Huttegger 2007a,b, forthcoming).

**3. Dynamics**

The simplest dynamic model of differential reproduction for a large population is the *replicator dynamics* (Taylor & Jonker 1978; Hofbauer & Sigmund 1998). Replicator dynamics has an alternative interpretation as a model of cultural evolution by imitation of successful strategies (Björnerstedt and Weibull 1995; Schlag 1998). It has a third interpretation as a limiting case of reinforcement learning (Beggs 2005; Hopkins & Posch 2005).

We can consider a single population models where strategies are conditional (if sender do this, if receiver do that), or a two population model with one population of senders and another population of receivers. Both have biological applications. A two population model is clearly appropriate for interspecies signaling. In the case same species alarm calls, individuals are sometimes in the role of sender and sometimes that of receiver.

For a single population, let the strategies be {S_i_}, let x_i_ be the population proportion of those who use strategy S_i_ and let the fitness of strategy S_i_ played against S_j_ be denoted W(S_i_|S_j_). Then, assuming random matching, the average fitness of strategy S_i_ is:

W(S_i_) = ∑_j_ x_j_ W(S_i_|S_j_)

and the average fitness of the population is:

W(S) = ∑_i_ W(S_i_) x_i_

The replicator dynamics is the system of differential equations:

dx_i_/dt = x_i_ [W(S_i_)-W(S)]

For the two-population case, let x_i_ be the population proportion of those who use strategy S_i_ in the population of senders and y_i_ be the population of those who use strategy R_i_ in the population of receivers. We again assume random matching of senders and receivers, so that:

W(S_i_) = ∑_j_ y_j_ W(S_i_|R_j_) and W(R_j_) = ∑_i_ x_i_ W(R_j_|S_i_)

The average fitnesses of the sender and receiver populations respectively are:

W(S) = ∑_i_ W(S_i_) x_i_ and W(R) = ∑_j_ W(R_j_) y_i_

We consider the evolution of this two population system using bipartite replicator dynamics (Taylor & Jonker 1978; Hofbauer & Sigmund 1998):

dx_i_/dt = x_i_ [W(S_i_)-W(S)]

dy_j_/dt = y_j_ [W(R_j_)-W(R)]

In both the single population and two-population models of Lewis’ signaling games, the strong common interest between sender and receiver assures *global convergence* of the replicator dynamics; all trajectories must lead to dynamic equilibria

(Hofbauer & Sigmund 1998; Huttegger 2007a,b).

In the case of a 2by2by2 Lewis signaling game, with states equiprobable, the “hasty conclusion” from evolutionarily stability equilibrium analysis is, in fact, born out by the dynamics. Equilibria other than the signaling systems are all dynamically unstable. In both two-population and one-population models, replicator dynamics carries almost all possible population proportions to a signaling system (Huttegger 2007a,b, forthcoming; Hofbauer & Huttegger 2008 ).

But if states are not equiprobable, this is no longer so. Suppose that state 2 is much more probable than state 1. Then the receiver might just do the act that is best in state 2 and ignore the signal. And since the signal is being ignored, the sender might as well ignore the state. Consider a population in which receivers always do act 2, some senders always send signal 1 and some senders always send signal 2. Any such population is an equilibrium. We have described a set of polymorphic pooling equilibria. These equilibria are dynamically stable, even though they are not evolutionarily stable in the sense of (Maynard-Smith and Price, 1973). They are not strongly stable attractors in the dynamics. Rather, they are “neutrally stable” in that points near them stay near them under the action of the dynamics. But they do not attract all points near them. For instance other pooling equilibria near them are not moved at all by the dynamics. The question is whether this set of pooling equilibrium, considered as a whole, has a basin of attraction. It has been shown analytically that it does (Hofbauer and Huttegger 2008). Simulations show that the size of the basin of attraction need not be negligible. The size depends, as would be expected, on the difference in probabilities of the two states. If we were to depart from the assumption that the states have equal payoffs, it would also depend on the magnitudes of the payoffs.

Even if we keep the states equiprobable and the magnitudes of the payoffs equal, almost sure convergence to a signaling system is lost of we move from 2by2by2 to 3by3by3. In this game, total pooling equilibria are dynamically unstable, but there are sets of neutrally stable partial pooling equilibria like the ones discussed in the last section. It can be shown analytically that the set of partial pooling equilibria has a positive basin of attraction, and simulation shows that this basin is not negligible. (Huttegger, Skyrms, Smead and Zollman 2006).

Even with the strong common interest assumptions built into Lewis’ signaling games, the emergence of signaling is not quite the sure thing that it may initially have seemed on the basis of equilibrium considerations. Perfect signaling systems can evolve, but it is not guaranteed that they will do so. Dynamic analysis has revealed unexpected subtleties.

There are more subtleties to explore, because the sets of suboptimal equilibria are not *structurally stable* (Guckenheimer and Holmes 1983; Skyrms 1999) Small perturbations of the dynamics can make a big difference. The natural perturbation to pure differential reproduction that needs to be considered is the addition of a little mutation. We can move from the replicator dynamics to the replicator-mutator dynamics (Hadeler 1981: Hofbauer 1985). For a two-population model with uniform mutation this is:

dx_i_/dt = x_i_ [(1-e)W(S_i_)-W(S)] + (e/n)W(S)

dy_j_/dt = y_j_ [(1-e)W(R_j_)-W(R)] + (e/n)W(R)

where e is the mutation rate and n is the number of strategies. We include all possible strategies. Evolutionary dynamics is now governed by a sum of selection pressure and mutation pressure. Mutation pressure pushes towards all strategies being equiprobable, where mutation into a strategy would equal mutation out. Mutation pressure can be counterbalanced or overcome by selection pressure. But if selection pressure is weak or non-existent, mutation can cause dramatic changes in the equilibrium structure of the interaction.

We can illustrate by returning to the 2by2by2 signaling game, two populations, states with unequal probability. Suppose state 2 is more probable than state 1. Then, as we have seen, there is a set of pooling equilibria for the replicator dynamics. In the receiver population, the strategy of always doing act 2 (no matter what the state) goes to fixation. In the sender population there is a polymorphism between two types of sender. One sends signal 1, no matter what the state; the other sends signal 2, no matter what the state. Since there is no selection pressure between the senders’ types, every such sender polymorphism is an equilibrium. Addition of *any* amount of uniform mutation leads the set of pooling equilibria to collapse to a single point at which “Always send signal 1” and “Always send signal 2” are represented with equal probability. (Hofbauer & Huttegger 2008) But all other strategies are also present in small amounts at this population state, due to the action of mutation.

The big question concerns the stability properties of this *perturbed pooling equilibrium.* Is it dynamically stable or unstable? There is no unequivocal answer. It depends on the disparity in probability between the two states (Hofbauer & Huttegger 2008). A little mutation can help the evolution of signaling systems, but does not always guarantee that they evolve.

**4. Costs**

Let us return to the case of 2by2by2, states equiprobable, but assume that *one of the signals costs something to send, while the other is cost-free*. (We could interpret the cost-free signal as just keeping quiet.) Now there are pooling equilibria in which the sender always sends the cost-free signal and there are various proportions of receiver types.

Denoting the sender’s strategies as:

*Sender 1*: State 1 => Signal 1, State 2 => Signal 2

*Sender 2*: State 1 => Signal 2, State 2 => Signal 1

*Sender 3*: State 1 => Signal 1, State 2 => Signal 1

*Sender 4*: State 1 => Signal 2, State 2 => Signal 2

and the receiver’s strategies as:

*Receiver 1*: Signal 1 => Act 1, Signal 2 => Act 2

*Receiver 2*: Signal 1 => Act 2, Signal 2 => Act 1

*Receiver 3*: Signal 1 => Act 1, Signal 2 => Act 1

*Receiver 4*: Signal 1 => Act 2, Signal 2 => Act 2

If signal 1 is costly, cost = 2c, states equiprobable, and a background fitness is 1, we have the payoff matrix (sender’s payoff, receiver’s payoff), as shown in table 1.

|  | Receiver 1 | Receiver 2 | Receiver 3 | Receiver 4 |
| --- | --- | --- | --- | --- |
| Sender 1 | **2-c, 2** | 1-c, 1 | 1.5-c, 1.5 | 1.5-c, 1.5 |
| Sender 2 | 1-c, 1 | **2-c, 2** | 1.5-c , 1.5 | 1.5-c, 1.5 |
| Sender 3 | 1.5-2c, 1.5 | 1.5-2c, 1.5 | 1.5-2c., 1.5 | 1.5-2c, 1.5 |
| Sender 4 | 1.5, 1.5 | 1.5, 1.5 | **1.5, 1.5** | **1.5, 1.5** |

Table 1

Sender’s strategy 1 and 2 pay the cost half the time, strategy 3 all the time, and strategy 4 never. Pure Nash equilibria of the game for small c are boldfaced.(If c>.5 it is never worth the cost to send a signal, and the signaling system equilibria disappear.) There is also a large range of mixed strategies (corresponding to receiver polymorphisms) that are equilibria. States when receiver types are approximately equally represented and senders always send the costless signal, are such pooling equilibria.

It might also *cost the receiver something to listen*. Let us combine this with a costly message and unequal state probabilities. For example, let the probability of state 1 be 1/3, the cost of signal 1 .3, and the cost of the receiver paying attention to the signals .1. The background fitness is 1. Then the foregoing payoff matrix changes to that displayed in table 2.

|  | Receiver 1 | Receiver 2 | Receiver 3 | Receiver 4 |
| --- | --- | --- | --- | --- |
| Sender 1 | **2-.1, 2-.1** | 1-.1, 1-.1 | 1.33-.1, 1.33 | 1.67-.1, 1.67 |
| Sender 2 | 1-.2, 1-.1 | **2-.2, 2-.1** | 1.33-.2, 1.33 | 1.67-.2, 1.67 |
| Sender 3 | 1.5-.3, 1.5-.1 | 1.5-.3, 1.5-.1 | 1.33-.3, 1.33 | 1.67-.3, 1.67 |
| Sender 4 | 1.5, 1.5-.1 | 1.5, 1.5-.1 | 1.33, 1.33 | **1.67, 1.67** |

Table 2

The *pooling equilibrium*, <sender 4, receiver 4>, where sender always sends signal 2 and receiver always does act 2, is now a *strict* Nash equilibrium of the game. Either sender or receiver who deviates does strictly worse. Thus, in both one and two population evolutionary models, it is *evolutionarily stable* and a strong (attracting) equilibrium in the replicator dynamics.

*If costs are state-specific,* a rosier picture is possible (Zahavi 1975). We alter the previous example so that signal 1 is free in state 1 but costs .3 in state 2 and signal 2 is free in state 2 but costs .3 in state 1. Sender 1 now pays no penalty; sender 2 always pays .3; sender 3 pays .3 two-thirds of the time (=.2); and sender 4 pays .3 one-third of the time (=.1). This is shown in table 3.

|  | Receiver 1 | Receiver 2 | Receiver 3 | Receiver 4 |
| --- | --- | --- | --- | --- |
| Sender 1 | **2, 2-.1** | 1, 1-.1 | 1.33, 1.33 | 1.67, 1.67 |
| Sender 2 | 1-.3, 1-.1 | **2-.3, 2-.1** | 1.33-.3, 1.33 | 1.67-.3, 1.67 |
| Sender 3 | 1.5-.2, 1.5-.1 | 1.5-.2, 1.5-.1 | 1.33-.2, 1.33 | 1.67-.2, 1.67 |
| Sender 4 | 1.5-.1, 1.5-.1 | 1.5-.1, 1.5-.1 | 1.33-.1, 1.33 | 1.67-.1, 1.67 |

Table 3

The pooling state, <Sender 4, Receiver 4>, is no longer an equilibrium at all. Given that the receiver is ignoring the message, the sender is better off switching to the costless strategy, Sender 1. If so, the receiver is better off switching to Receiver 1, yielding the optimal signaling system <Sender 1, Receiver 1>. Optimality, however, may not evolve. The *suboptimal signaling system* <Sender 2, Receiver 2>, in which the sender uses the “wrong” signals and always pays a signaling cost, is also a strict equilibrium. Both signaling systems are strong (attracting) equilibria in both one and two population replicator dynamic models.

**5. Signaling Networks**

There is no reason to limit ourselves to signaling between just two actors, one sender and one receiver. In fact, most signaling systems in nature involve multiple senders, or multiple receivers, or both. If a receiver gets signals carrying different pieces of information from different senders, the signaling system is called upon to solve some problem of information processing. Consider a toy model with two senders and one receiver:

•→•←•

*Signaling Complementary Information*. There are four states of nature, each of which occurs with non-zero probability. Two individuals are situated so as to make different incomplete observations of the state. The first sees whether it is in {S1, S2} or in {S3, S4} and the second sees whether it is in {S1, S3} or in {S2, S4}. Together they have enough information to pin down the state of nature, but separately they do not. Each sends one of two signals to a receiver who must choose one of four acts. Let’s say the first send chooses “red” or “green” and the second chooses “blue” or “yellow.” The payoffs favor cooperation. Exactly one act is “right” for each of the states in that each of the individuals is reinforced just in case the “right” act for the state is chosen.

In this extended Lewis signaling game the observational situation of sender 1 is characterized by a partition of the states, O_1_ = {{S1, S2}, {S3, S4}}. Her signaling strategy is a function from the elements of this partition into her set of signals, {R, G}. Likewise sender 2 in observational situation O_2_ = {{S1, S3}, {S2, S4}} has a signaling strategy that maps the elements of her partition into her signal set, {B, Y}. The receiver’s strategy maps pairs of signals {{R, B}, {R, Y}, {G, B}, {G, Y}} into her set of acts {A1, A2, A3, A4}.

All agents get payoff 1 just in case the receiver correctly identifies the state and does the appropriate act. Payoffs are shown in table 4.

|  | Act 1 | Act 2 | Act 3 | Act 4 |
| --- | --- | --- | --- | --- |
| State 1 | 1,1,1 | 0,0,0 | 0,0,0 | 0,0,0 |
| State 2 | 0,0,0 | 1,1,1 | 0,0,0 | 0,0,0 |
| State 3 | 0,0,0 | 0,0,0 | 1,1,1 | 0,0,0 |
| State 4 | 0,0,0 | 0,0,0 | 0,0,0 | 1,1,1 |

Table 4

A *signaling system* equilibrium is a combination of sender and receiver strategies such that payoff is equal to one in each state. As before, a signaling system is a *strict equilibrium* of the game, and signaling systems are the *only* strict equilibria. There are lots of pooling and partial pooling equilibria.

In an evolutionary setting, this three-player game gives rise to three-population models, two-population models, and one population models. In a one-population model, an individual’s strategy would be of the form: *If sender in observational situation O_1_ have this sender’s strategy, if sender in observational situation O_2_ have that sender’s strategy; if receiver have this strategy.* The most natural two population model has a population of senders with different observational roles and a population of receivers. In all three evolutionary settings signaling systems are the unique evolutionarily stable states. It is no longer certain that a signaling system must evolve, but it is certain that a signaling system *can* evolve. In each of these settings a signaling system is a strongly stable (attracting) equilibrium in the replicator dynamics.

Each sender’s signal conveys perfect information about her observation – about the partition of states of the world that she can see. The combination of signals has perfect information about the states of the world. Exactly one state corresponds to each combination of signals. And the receiver puts the signals together. The receiver’s acts contain perfect information about the state of the world. *The signaling system simultaneously solves problems of transmission and integration of information.*

The basic model admits of interesting variations. Of course there may be more senders. And depending on the act set available to the receiver, he may draw the appropriate logical “conclusion” from the “premises” supplied by the various senders. (Skyrms 2000, 2004, 2008) The senders’ partitions may not be fixed by nature, but may themselves evolve in the presence of information bottlenecks (Barrett 2006, 2007a,b).

*Error:* There is another class of multiple sender models, where the question is not one of complementary information but one of error. In the previous example, senders observed different partitions but there was no error in identifying the true element of the partition. Here we suppose that the senders all observe the same states but with some error in correctly identifying them. (An alternative, essentially equivalent, interpretation of the model would locate the errors in the transmission of the signals.)

For the simplest model, suppose that there are only two states and two acts. States are equiprobable. Three senders observe the states with error probability of 10%, with the errors being independent between senders and between trials. Each sender sends a message to the receiver, who must then choose one of the two acts. As before, we assume that act one pays off 1 for everyone involved in state 1 and act 2 pays of 1 for everyone in state 2. Otherwise no one gets anything.

Nature here first flips a coin to pick a state, and then picks *apparent states* to present to the three senders according to the error probabilities. A sender’s strategy is a function from apparent state into the set of signals, {S1, S2}. We have a choice about how to set up the receiver’s strategies. If we were to assume that the receiver could distinguish between senders, we could take the receiver’s strategy to be a function from ordered triples of signals to acts. But here we assume that the receiver cannot distinguish between <S1, S2, S1>, <S1, S1, S2> and <S1, S1, S2>. The receiver here has an observational partition and can only count signals. This might be thought of as discrete approximation to a situation where the receiver perceives an intensity arising from many chemical signals, or the sound intensity arising from many calls. A receiver’s strategy is then a function from frequencies of signal received to act.

Optimal signaling in this model consists in what we might call a *Condorcet equilibrium* (see List et. al. this issue, and Sumpter and Pratt this issue.) There is one signal that the senders all use for apparent state 1 and another that they all use for apparent state 2. The receiver goes with a majority vote. For instance, if the senders all send signal 2 in state 1, the receiver will do act 2 if two or more senders send signal 2 and act 1 otherwise. In our example, individuals at a Condorcet equilibrium reduce their error rate from 10% to under 3%. This can be viewed as an example of information filtering, as explained in the introduction.

Rather that thinking of evolution taking place solely in the context of this game, we might assume that sender’s strategies already evolved in the context of single sender-receiver interactions. Then receivers usually get one signal, or multiple agreeing signals according to the evolved signaling system, but occasionally get disagreeing signals. Slow adaptation for mixed signals in such an environment is a simple problem of optimization.

Against these fixed sender strategies, receivers who go with the majority of senders will have the greatest fitness. Then replicator dynamics will converge to the optimal receiver strategy (Hofbauer and Sigmund 1998).

But suppose we forego this easy route and ask whether Condorcet signaling equilibria can evolve in the context of the original four-person game. Both the sender’s signals and the receiver’s voting rule must co-evolve. It is still possible for efficient signaling to evolve. Condorcet equilibria are strict. Consequently they are stable attractors in evolutionary versions of this game using replicator dynamics. In fact, simulations show Condorcet equilibria almost always evolving in the foregoing model

(See supporting matter).

Variations of the parameters of the model may well lead to the evolution of voting rules different from majority rule. This is an area open for exploration. Recent rational-choice literature on strategic voting (Austen-Smith and Banks 1996; Feddersen and Pesendorfer 1998) is a source of a rich set of models that can be transposed to an evolutionary setting.

*Teamwork*: It is sometimes the case that a well-placed sender knows what needs to be done, and can send messages to receivers who can act, but that no one receiver can do everything that needs to be done. The sender may be the foreman, or the commander, or the brain of an organism – the team leader. Success for all requires teamwork.

There may be one sender and multiple receivers:

•←•→•

For a simple teamwork problem, we suppose that there are two receivers and one sender. The sender observes one of four equiprobable states of the world and sends one of two signals to each receiver. The receivers must each choose between two acts, and the acts must be coordinated in a way determined by the state for all to get a payoff. We take payoffs to be as in table 5.

|  | <A1, A1> | <A1, A2> | <A2, A1> | <A2, A2> |
| --- | --- | --- | --- | --- |
| State 1 | 1,1,1 | 0,0,0 | 0,0,0 | 0,0,0 |
| State 2 | 0,0,0 | 1,1,1 | 0,0,0 | 0,0,0 |
| State 3 | 0,0,0 | 0,0,0 | 1,1,1 | 0,0,0 |
| State 4 | 0,0,0 | 0,0,0 | 0,0,0 | 1,1,1 |

Table 5

We assume that the sender can distinguish members of the team, so sender’s strategy maps states into ordered pairs of signals and a receiver’s strategy maps her signal into her space of acts. Here the problem to be solved is a combination of one of communication and one of co-ordination. It is solved in a signaling system equilibrium, in which everyone always gets payoff of one. A signaling system equilibrium is again a strict equilibrium, and the unique strict equilibrium in the game. It is a strongly stable attractor in the replicator dynamics.

The example can be varied in many ways, some more interesting than others. The two receivers can be thought of as playing a rather trivial two-person game, but the game is different in every state of the world. In a signaling system, the sender can be thought of either as conveying information about the game or about the optimal act to be done. In these trivial games, these are equivalent. The example could be varied by changing the four embedded two-person games and their effect on the payoffs to the sender.

*Chains*: Information can flow further than shown in the models given so far. Signalers can form chains, so that information is passed along until it reaches an endpoint at which it can be used. Consider a little signaling chain.

•→•→•

There are a sender, an intermediary, and a receiver. Nature chooses one of two states with equal probability. The sender observes the state, chooses one of two signals and sends it to the intermediary, the intermediary observes the sender’s signal, chooses one her own two signals, and sends it to the receiver. The receiver observes the intermediary’s signal and chooses one of two acts. If the act matches the state, sender, intermediary and receiver all get a payoff of one, otherwise a payoff of zero.

Suppose that the set of potential signals available to the sender is {R, B}, and that available to the receiver is {G, Y} A sender’s strategy is a function from {S1, S2} into {R, B}, and intermediary’s from {R, B} into {G, Y}, and a receiver’s from {G, Y} into {A1, A2}. A signaling system here is a triple of strategies such that the composition of sender’s strategy, intermediary’s strategy, receiver’s strategy, maps state 1 to act 1 and state 2 to act 2. Signaling systems are the unique strict equilibria in this game and the unique evolutionarily stable states in the corresponding one, two and three population signaling games. They are attractors in the replicator dynamics. In principle, signaling chains can evolve out of nothing.

However, simulations show that in this case evolution is very slow when compared with the other signaling games discussed so far. This may simply be a consequence of the multiplicity of coordination problems that need to be solved simultaneously. The speed with which the chain signaling system can evolve is much improved if the sender and receiver have pre-existing signaling systems. They could be the same signaling system, which would be plausible if sender and receiver were members of the same population, but the signaling systems need not be the same. Sender and receiver can have different “languages” so that the intermediary has to act as a “translator”, or signal transducer. Suppose that the sender sends Red or Blue and the ultimate receiver reacts to Green or Yellow as follows:

SENDER RECEIVER

State 1 => R G => Act 2

State 2 => B Y =>Act 1

A successful translator must learn to receive one signal and send another, so that the chain leads to a successful outcome.

SENDER TRANSLATOR RECEIVER

State 1 => R see R => send Y Y => Act 1

State 2 => B see B => send G G => Act 2

The translator’s learning problem is now really quite simple. The requisite strategy strictly dominates all alternatives. It pays off all the time, while the strategies *Always send Y* and *Always send G* pay off half the time, and the remaining possibility always leads to failure. The dominated strategies are eliminated (Hofbauer and Sigmund 1998), and the correct strategy evolves.

*Dialogue*: The chain model showed one way in which simple interactions could be strung together to form more complex signaling systems. Here is another. Suppose that a sender’s observational partition is not fixed. The sender can choose which observation to make. That is to say, she can choose which partition of states to observe. Suppose also, that the receiver’s decision problem is not fixed. Nature chooses a decision problem to present to the receiver. Different sorts of information are relevant to different decision problems. Knowing the actual element of partition A (the element that contains the actual state) may be relevant to decision problem 1, while knowing the actual element of partition B may be relevant to decision problem 2. This opens up the possibility of signaling dialogue, where information flows in two directions:

•↔•

In the simplest sort of example, nature flips a coin and presents player 2 with one or another decision problem. Player 2 sends one of two signals to player 1. Player 1 selects one of two partitions of the state of nature to observe. Nature flips a coin and presents player 1 with the true state. Player 1 sends one of two signals to player 2. Player 2 chooses one of two acts.

Suppose that there are four states, {S1, S2, S3, S4}, with alternative partitions:

P1 = {{S1, S2}, {S3, S4}}, P2 = {{S1, S3}, {S2, S4}}. The two decision problems require choices in different act sets: D1 = {A1, A2}, D2 = {A3, A4}. Payoffs for the two decision problems are shown in table 6.

|  | *Decision 1* | *Decision 1* | *Decision 2* | *Decision 2* |
| --- | --- | --- | --- | --- |
|  | Act 1 | Act 2 | Act 3 | Act 4 |
| State 1 | 1 | 0 | 1 | 0 |
| State 2 | 1 | 0 | 0 | 1 |
| State 3 | 0 | 1 | 1 | 0 |
| State 4 | 0 | 1 | 0 | 1 |

Table 6

Player 2 has a signal set {R, G} and player 1 has a signal set {B, Y}. A strategy for player 2 now consists of three functions, one a sender strategy from {P1, P2} into {R, G}, one a receiver strategy form {B,Y} into {A1, A2}, one a receiver strategy from {B, Y} into {A3, A4}. In a signaling system equilibrium each player gets always gets a payoff of one. The possibility of dialogue introduces a plasticity of signaling that is absent in fixed sender-receiver games. Signaling systems are strict, and evolutionarily stable as before.

Signaling systems can evolve in the dialogue interaction in isolation, but simulations show this process to be very slow. As in the case of chains, evolution of a signaling system is much easier if we assume that some of its parts have evolved in less complicated interactions. Player one may already have signaling systems in place for the two different observational partitions as a consequence of evolution in simple sender-receiver interactions. If so, the evolution of dialogue only requires that the second player signal the problem and the first choose what to observe. This is no more difficult than evolution of a signaling system in the original Lewis signaling game.

**6. Conclusion**

We have investigated the evolution of signaling in some modest extensions of Lewis signaling games with multiple senders and receivers. This discussion has focused on one particular setting – a large (infinite) population or several large populations with random interactions between individuals. Different settings would call for different relevant dynamics. A small population with random encounters calls for a stochastic model of evolution, with either a growing population or one whose size is fixed at some carrying capacity (Shreiber 2001; Benaim, *et al.* 2004; Taylor, Fudenberg, Sasaki and Nowak 2004). Pawlowitsch (2007) shows that in one kind of finite population model, efficient proto-languages are the only strategies that are *protected by selection.* Individuals might interact with neighbors in some spatial structure (Grim *et al.* 2002; Zollman 2005). Isolated individuals might invent signaling systems by trial and error learning in repeated interactions. (Skyrms 2004, 2008; Barrett 2004, 2007a,b), which might then spread by a process of cultural evolution (Komarova & Niyogi 2004). In fact, urn models of reinforcement learning are very close to urn models of evolution in a small, growing population (Shreiber 2001; Benaim, *et al.* 2004). It has been recently proved that reinforcement dynamics in the simplest Lewis signaling game - 2by2by2 states equiprobable – converges with probability one to a signaling system (Argiento *et al.* 2008). Such an analytic treatment of reinforcement learning does not yet exist for more complicated signaling interactions, but simulations tend to give results parallel to the evolutionary analysis given here. This is not entirely surprising, given the close connections between reinforcement learning and the replicator dynamics (Beggs 2005; Hopkins & Posch 2005).

Simple models such as those discussed here can be assembled into more complex and biologically interesting systems. The network topologies themselves may evolve. (Skyrms and Pemantle 2000; Bala and Goyal 2000). There are all sorts of interesting variations. For instance, signaling networks may allow eavesdroppers, a case well-studied in (McGregor 1995). But the main business of signaling networks is to facilitate successful collective action. The simple models studied here focus on crucial aspects of coordinated action. Information is acquired by units of the group. It is transmitted to other units and processed in various ways. Extraneous information is discarded. Various kinds of computation and inference are performed. The resulting information is used to guide group decisions that lead to coordinated action. All this can happen either with or without conscious thought. These processes are instantiated in human organizations, in the co-ordination of the organs and cells of a multicellular organism, and even within the cells themselves. Information flows through signaling networks at all levels of biological organization.

I would like to thank Jeffrey Barrett, Simon Huttegger, Louis Narens, Don Saari, Rory Smead, Elliott Wagner, and Kevin Zollman for many discussions. Rory Smead performed the “Taking as Vote” simulations reported in the supporting matter. I would also like to thank two anonymous referees for this journal who provided many helpful suggestions for improvement.

**References**

Argiento, R., Pemantle, R., Skyrms, B. and Volkov, S. 2008 Learning to Signal: Analysis of a Micro-Level Reinforcement Model. *Stochastic Process. Appl.* ([doi:10.1016/j.spa.2008.02.014](http://dx.doi.org/10.1016/j.spa.2008.02.014) ).

Austen-Smith, D. and Banks, J. S. 1996 Information Aggregation, Rationality, and the Condorcet Jury Theorem. *Am. Pol. Sci. Rev.* **90**, 34-45.

Bala, V. and Goyal, S. 2000 A Non-Cooperative Model of Network Formation. *Econometrica* 1181-1229.

Barrett, J. A. 2006 Numerical Simulations of the Lewis Signaling Game: Learning Strategies, Pooling Equilibria, and the Evolution of Grammar. Working Paper MBS06-09. University of California, Irvine.

Barrett, J. A. 2007a The Evolution of Coding in Signaling Games” *Theory Dec.*.

(doi:10.1007/s11238-007-9064-0).

Barrett, J. A. 2007b Dynamic Partitioning and the Conventionality of Kinds. *Philos. Sci.* **74**, 527-546*.*

Beggs, A. 2005 On the Convergence of Reinforcement Learning. *J. Econom. Theory* **122**, 1-36.

Benaim, M., Shreiber, S. J. and Tarres, P. 2004 Generalized Urn Models of Evolutionary Processes. *Ann. Appl. Probab.* **14**, 1455-1478.

Björnerstedt, J. and Weibull, J. 1995 Nash Equilibrium and Evolution by Imitation. In *The Rational Foundations of Economic Behavior* ed. K. Arrow *et* *al*. 155-171 New York: MacMillan.

Charrier, I. and Sturdy, C. B. 2005 Call-Based Species Recognition in the Black-Capped Chickadees. *Behav. Process.* **70**, 271-281.

Cheney, D. and Seyfarth, R. 1990 *How Monkeys See the World: Inside the Mind of Another Species.* Chicago: University of Chicago Press.

Dyer, F. C. and Seeley, T. D. 1991 Dance Dialects and Foraging Range in three Asian Honey Bee Species. *Behav. Ecol. Sociobiol.* **28**, 227-233.

Evans, C. S., Evans, C. L. and Marler, P. 1994 On the Meaning of Alarm Calls: Functional Reference in an Avian Vocal System. *Anim. Behav.* **73**, 23-38.

Feddersen, T. and Pesendorfer, W. 1998 Convicting the Innocent: The Inferiority of Unanimous Jury Verdicts under Strategic Voting. *Am. Pol. Sci. Rev.* **92**, 23-35.

Green, E. and Maegner, T. 1998 Red Squirrels, *Tamiasciurus hudsonicus,* produce predator-class specific alarm calls. *Anim. Behav.* **55**, 511-518.

Grim, P., St. Denis, P., and Kokalis, T. 2002 Learning to Communicate: The Emergence of Signaling in Spatialized Arrays of Neural Nets. *Adap. Behav.* **10**, 45-70.

Gyger, M., Marler, P. and Pickert, R. 1987 Semantics of an Avian Alarm Call System: the Male Domestic Fowl, *Gallus Domesticus*. *Behavior* **102**, 15-20.

Guckenheimer, J. and Holmes, P. 1983 *Nonlinear Oscillations, Dynamical Systems, and Bifurcations of Vector Fields.* New York: Springer.

Hadeler, K. P. 1981 Stable Polymorphisms in a Selection Model with Mutation. SIAM J. Appl. Math. **41**, 1-7.

Hailman, J., Ficken, M. and Ficken, R. 1985 The ‘chick-a-dee’ calls of *Parus atricapillus* *Semiotica* **56**, 191-224.

Hofbauer, J. 1985 The Selection-Mutation Equation. *J. Math. Biol.* **23**, 41-53.

Hofbauer, J. and Huttegger, S. M. 2008 Feasibility of Communication in Binary Signaling Games. *J. Theor. Biol.* (doi:10.1016/j.jtbi.2008.07.010).

Hofbauer, J. and Sigmund, K. 1998 *Evolutionary Games and Population Dynamics.* Cambridge: Cambridge University Press.

Hopkins, E. and Posch, M. 2005 Attainability of Boundary Points under Reinforcement Learning. *Games Econom. Behav.* **53**, 110-125.

Huttegger, S. 2007a Evolution and the Explanation of Meaning. *Philos. Sci.* **74**, 1-27.

Huttegger, S. 2007b Evolutionary Explanations of Indicatives and Imperatives. *Erkenntnis* **66**, 409-436*.*

Huttegger, Simon (forthcoming) Robustness in Signaling Games. *Philos. Sci.* – special issue for PSA2006.

Huttegger, S., Skyrms, B., Smead, R. and Zollman, K. 2006 Evolutionary Dynamics of Lewis Signaling Games: Signaling Systems vs. Partial Pooling. Working Paper University of California, Irvine. (forthcoming in *Synthese*.)

Kaiser, D. 2004 Signaling in Myxobacteria. *Annu. Rev. Microbiol.* **58**, 75-98.

Komarova, N. and Niyogi, P. 2004 Optimizing the Mutual Intelligibility of Linguistic Agents in a Shared World. *Artificial Intelligence* **154**, 1-42.

Lewis, D. K. 1969 *Convention* Cambridge, Mass: Harvard University Press.

List, C., Elsholtz, C. and Seeley, T. D. This issue. Independence and Interdependence in Collective Decision Making: An Agent-Based Model of Nest-Site Choice by Honey Bee Swarms. *Philos. T. Roy. Soc. B.*

Manser, M., Seyfarth, R. M. and Cheney, D. 2002 Suricate Alarm Calls Signal Predator Class and Urgency. *Trends Cog. Sci.* **6**, 55-57

Maynard Smith, J. 1982 *Evolution and the Theory of Games*. Cambridge: Cambridge University Press.

Maynard Smith, J. and Price, G. 1973 The Logic of Animal Conflict. *Nature* **246**, 15-18.

McGregor, P. 2005 *Animal Communication Networks* Cambridge:Cambridge University Press.

Pawlowitsch, C. 2007 Finite Populations Choose an Optimal Language. *J. Theor. Biol.* **249**, 606-616.

Pawlowitsch, C. 2008 Why Evolution Does Not Always Lead to an Optimal Signaling System. *Games Econom. Behav.* **63**, 203-226.

Schauder, S. and Bassler. B. 2001 The Languages of Bacteria. *Genes Dev.* **15**, 1468- 1480.

Seyfarth, R. M. and Cheney, D. L. 1990. The Assessment by Vervet Monkeys of Their Own and Other Species’ Alarm Calls. *Anim. Behav.* **40**:754-764.

Schlag, K. 1998 Why imitate and if so, How? A Bounded Rational Approach to Many

Armed Bandits. *Journal Econom. Theory* 78, 130-156.

Shreiber, S. 2001 Urn Models, Replicator Processes and Random Genetic Drift. *SIAM J. Appl. Math.* 61: 2148- 2167.

Skyrms, B. 1996 *Evolution of the Social Contract* Cambridge: Cambridge University Press.

Skyrms, B. 1999 Stability and Explanatory Significance of Some Simple Evolutionary Models. *Philos. Sci.* 67, 94-113.

Skyrms, B. 2000 Evolution of Inference. In Dynamics of Human and Primate Societies (ed. T. Kohler and G. Gumerman), pp. 77-88. New York: Oxford University Press.

Skyrms, B. 2004 The Stag Hunt and the Evolution of Social Structure Cambridge: Cambridge University Press.

Skyrms, B. 2008 Signals. Presidential Address of the Philosophy of Science Association. Forthcoming in *Philos. Sci.*

Skyrms, B. and Pemantle, R. 2000 A Dynamic Model of Social Network Formation. *PNAS* 97, 9340-9346.

Sumpter, D. J. T. and Pratt, S. C. This issue. Quorum Responses and Consensus Decision-Making. *Philos. T. Roy. Soc. B.*

Taga, M. E. and Bassler, B. L. 2003 Chemical Communication Among Bacteria. *PNAS* 100 Suppl.2, 14549-14554.

Taylor, C. ,Fudenberg, D., Sasaki, A. and Nowak, M. 2004 *Bull. Math. Biol.* 66, 1621- 1644.

Taylor, P. and Jonker, L. 1978 Evolutionarily Stable Strategies and Game Dynamics. *Math. Biosci.* 40, 145-156.

Wärneryd, Karl 1993 Cheap Talk, Coordination, and Evolutionary Stability. *Games Econom. Behav.* 5, 532-546.

Zahavi, A. 1975 Mate Selection – Selection for a Handicap. *J. Theor. Biol.* 53, 205-214.

Zollman, K.2005 Talking to Neighbors: The Evolution of Regional Meaning. *Philos. Sci.* 72, 69-85.
